# Comparative Analysis of Modern 3D-Printed Hybrid Resin-Ceramic Materials for Indirect Restorations: An In Vitro Study

**DOI:** 10.3390/polym16223161

**Published:** 2024-11-13

**Authors:** Miriam Albrecht, Franziska Schmidt, Franziska Menzel, Jamila Yassine, Florian Beuer, Alexey Unkovskiy

**Affiliations:** 1 Department of Prosthodontics, Geriatric Dentistry and Craniomandibular Disorders, Charité—Universitätsmedizin Berlin, Aßmannshauser Str. 4–6, 14197 Berlin, Germany; franziska.schmidt2@charite.de (F.S.); franziska.menzel@charite.de (F.M.); jamila.yassine@charite.de (J.Y.); florian.beuer@charite.de (F.B.); alexey.unkovskiy@charite.de (A.U.); 2 Department of Dental Surgery, Sechenov First Moscow State Medical University, Bolshaya Pirogovskaya Street, 19c1, Moscow 119146, Russia

**Keywords:** CAD/CAM, additive manufacturing, subtractive manufacturing, biocompatibility, composite resins, artificial aging, thermocycling, color stability, surface roughness, SEM

## Abstract

The study investigated the impact of aging on surface roughness, color stability, and biocompatibility of hybrid resin-ceramic materials. A total of 225 specimens were produced from three three-dimensional (3D)-printed (HarzLabs Dental Sand Pro (HL), BEGO VarseoSmile Crown plus (BV), Voco V-Print c&b temp (VV)) and one milled material (Voco Grandio Blocs (VG)). Specimens were grouped into untreated, polished, and glazed surfaces. 5000 thermal cycles simulated aging. Surface roughness and color stability were analyzed, and surface topography was observed using scanning electron microscopy (SEM). Biocompatibility was evaluated with L929 cells. Surface roughness differed significantly between untreated and other groups, with no changes before and after artificial aging. Untreated milled samples were significantly smoother than 3D-printed ones. SEM analysis revealed roughest surfaces in untreated 3D-printed specimens. Polished and glazed specimens were smoother than untreated ones. Color values showed significant differences between untreated and treated/aged groups. No material showed cytotoxicity. In summary, untreated VG was smoother than 3D-printed materials, but polishing and glazing reduced roughness to levels comparable to VG. Surface treatments induced color changes, with glazing causing more changes than polishing. Aging affected color stability and biocompatibility but not surface roughness. All materials showed acceptable color changes and good biocompatibility.

## 1. Introduction

Computer-aided design (CAD) and computer-aided manufacturing (CAM) are still rapidly evolving technologies that have created the opportunity for the widespread application of digital technology in dentistry [1]. Compared to subtractive manufacturing, three-dimensional (3D) printing reduces material waste and allows complex geometries to be produced in a single step [2,3,4,5,6]. In addition, less waste of raw materials is associated with lower manufacturing costs [4,5]. Therefore, 3D printing has recently become an attractive alternative for the production of occlusal splints, dental models, surgical guides, and various dental restorations [2]. Three-dimensional printing also offers considerable interdisciplinary potential for complex dental and craniofacial applications for pediatric and elderly patients with facial defects [7,8]. This highlights the versatility and patient-specific adaptability of 3D-printed materials.

While various additive manufacturing (AM) techniques exist [1,2], resin-based materials are predominantly processed using digital light processing (DLP) printers [9,10]. DLP utilizes a digital micromirror and high-power LED sources to build up photopolymerizable liquid resin layer by layer to receive the desired 3D object [10,11]. The requirements for dental crown bridge resins include mechanical properties that can prevent failure under functional loading in the oral cavity and maintain color stability [2,12], as well as biological properties that can ensure biocompatibility [2,13]. A low surface roughness should be aimed for, as it is associated with lower stain adhesion and thus a longer-term esthetic appearance of the restoration, as well as lower bacterial adhesion [14]. Several studies have investigated the performance of 3D-printed and milled resins, yielding varying results [1,4,9,15,16]. Most of these studies concentrated on just one material type, as there was a limited offer on the market. Recently, further resin-ceramic materials indicated for permanent restorations have been introduced. For these reasons, the present study aims to investigate the influence of aging on the mechanical and biological properties of three DLP-printed and one milled hybrid resin-ceramic materials. The null hypothesis of the study was that there would be no difference between the 3D-printed and milled hybrid resin-ceramic materials in terms of biocompatibility, surface roughness, and color stability before and after aging.

## 2. Materials and Methods

**Figure 1:**
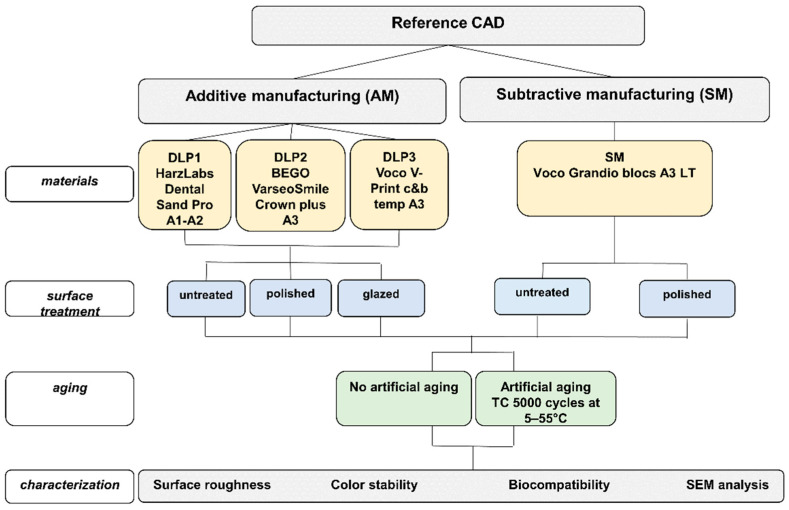
Study flowchart.

Figure 1 summarizes the entire experimental workflow.

### 2.1. Specimen Design and Manufacturing

Specimens were designed as small bars (12 × 9 × 2 mm) using CAD software (FreeCADSoftware, Version 0.20) and exported in a Standard Tesselation Language (STL) format. Table 1 lists the materials, and Table 2 shows the manufacturing and post-processing methods.

### 2.2. Surface Treatment

**Figure 2:**
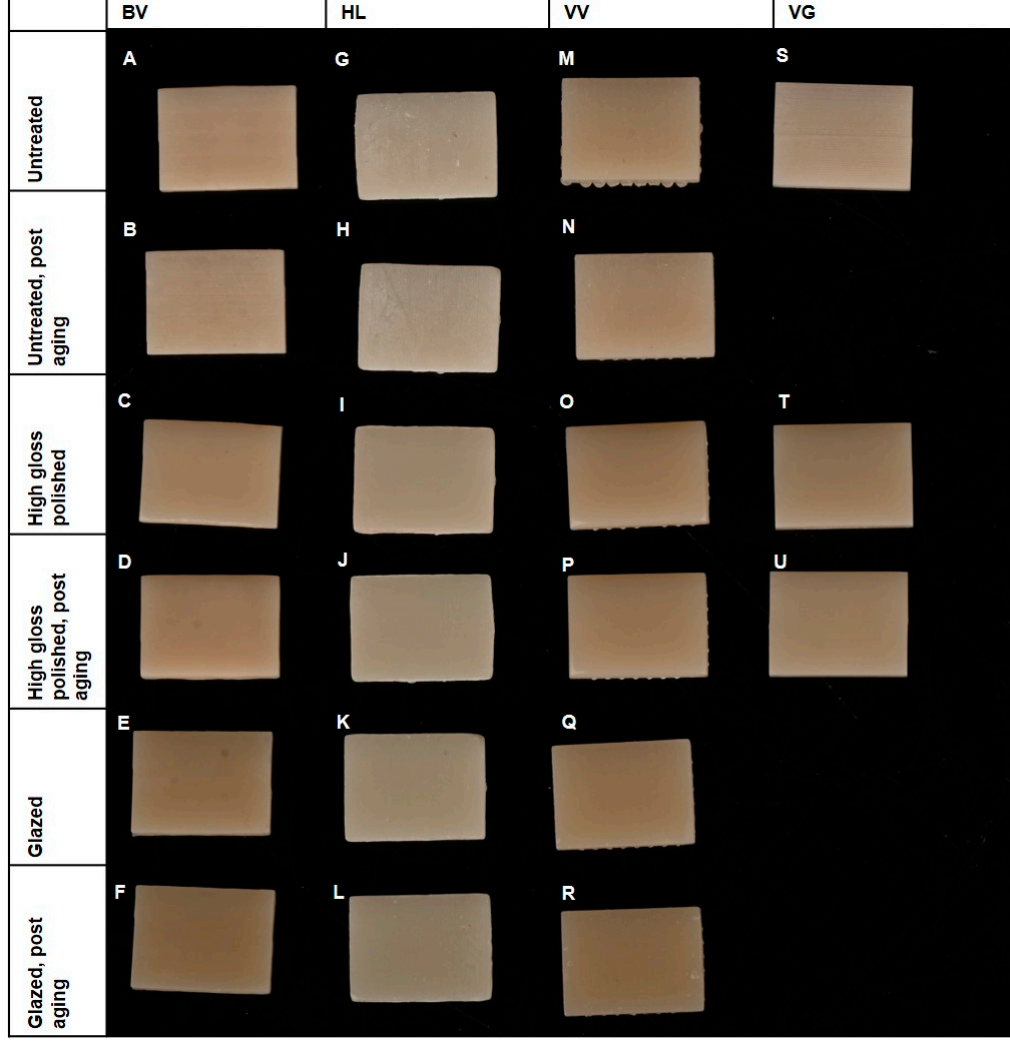
Comparative macroscopic photo of the samples. BV group: (**A**), untreated; (**B**), untreated, post-aging; (**C**), polished; (**D**), polished, post-aging; (**E**), glazed; and (**F**), glazed, post-aging. HL group: (**G**), untreated; (**H**), untreated, post-aging; (**I**), polished; (**J**), polished, post-aging; (**K**), glazed; and (**L**), glazed, post-aging. VV group: (**M**), untreated; (**N**), untreated, post aging; (**O**), polished; (**P**), polished, post aging; (**Q**), glazed; and (**R**), glazed, post aging. VG group: (**S**), untreated. (**T**), polished. (**U**), polished, post aging.

Specimens were divided into three groups according to the surface treatment: (1) untreated, (2) high gloss polished, following the polishing protocol of Kraemer et al. [18], and (3) glazed. Table 3 shows the number of specimens for each group, and Figure 2 shows a comparative macroscopic photo of the samples.

Specimens were first pre-polished with grinding paper (Corund SC 150, Hager & Werken, Duisburg, Germany), followed by abrasive paper with a grain size of 180. The grinding paper was inserted into the laboratory micromotor with a straight hand piece and control box. Secondly, the specimens were brushed (Abraso-Soft Acryl, Bredent, Senden, Germany) with the pumice slurry (Steribim super, BEGO, Bremen, Germany) on the brushing machine (Poliereinheit PE5, Degussa AG, Hanau, Germany). After using the rotary bristle brush, the specimens were polished with a soft cloth wheel (Polirapid, Dr. Montemerlo, Singen, Germany) and polishing paste (Universal Polishing Paste, Ivoclar Vivadent, Schaan, Liechtenstein) to achieve a high gloss.

One-third of the 3D-printed samples were thinly coated with a single layer of glaze: HL with HARZ Labs Glaze (HARZ Labs), BV with Optiglaze Color Transparent (GC Corporation, Tokyo, Japan), and VV with Easy Glaze (VOCO), according to the manufacturer’s instructions.

### 2.3. Thermocycling

To simulate aging, some samples underwent 5000 thermal cycles between 5 and 55 °C in distilled water in a thermocycling machine (JULABO GmbH, Seelbach, Germany) with a dwell time of 30 s in each bath. A total of 5000 thermal cycles have been reported to be equivalent to 6 months of clinical use [19].

### 2.4. Surface Roughness

Surface roughness was measured using an optical roughness measuring microscope (Infinite Focus G4, Alicona Imaging, Raaba/Graz, Austria). Each sample was scanned at ×20 magnification under standard light conditions, and 3D image viewer software (Alicona, Raaba/Graz, Austria) was used for data analysis. An area of 0.71 × 0.54 mm was recorded and filtered through a Gaussian filter (0.8 mm). The average profile depth (R_a_) was calculated using the 3D images obtained along a 4 mm line. Three different measurements were taken for each sample. The average of these measurements was calculated. The average R_a_ values were used for statistical analysis.

### 2.5. Color Stability

Color values of the samples were recorded with a calorimeter (X-Rite Ci7600, X-Rite with Color iQC10 software, Grand Rapids, MI, USA). The digital spectrophotometer had an aperture size of 6 mm and a D65 illumination curve. Before testing, calibrations were performed according to the manufacturer’s instructions. Color parameters of each sample were measured against a white background. Each sample was measured in reflectance mode against the same background in five different areas of the sample: the center, right center edge, left center edge, top center edge, and bottom center edge. The CIE L* a* b* system was used in conjunction with the calorimeter for quantitative measurements. The coordinates L* (lightness and differences between darkness and brightness), a* (differences in the red–green axis), and b* (differences in the yellow–blue axis) of the samples were obtained. ΔL*, Δa*, and Δb* are the differences in the color parameters L*, a*, and b* between the mean values of the measurements of untreated samples before artificial aging and the values of samples of the same material with surface treatment and/or thermocycling. The color differences (ΔEcmc) were calculated according to the CIE ΔEcmc formula [20]:

(1) $$\Delta \mathrm{Ecmc}=\sqrt{{\left(\frac{\Delta L}{lS_{L}}\right)}^{2}+{\left(\frac{\Delta C}{cS_{c}}\right)}^{2}+{\left(\frac{\Delta H}{S_{H}}\right)}^{2}}$$

### 2.6. Biocompatibility

To investigate biocompatibility by indirect contact, specimens were disinfected with 70% ethanol and UV-sterilized for 30 min before preparation of the extracts according to DIN EN ISO 10993-12 [21]. The cell culture medium for L929 cells consisted of 88% RPMI (Roswell Park Memorial Institute 1640 Medium, gibco/Thermo Fisher Scientific, Waltham, MA, USA), 10% Fetal Bovine Serum (FBS), 1% MEM Non-Essential Amino Acid Solution and 1% Penicillin/Streptomycin. After 24 h at 37 °C and 5% CO_2_ in the incubator, L929 cells were seeded (cell harvest and cell counting of thawed and subcultured cells—1.09 × 104 cells per well in a 96-well plate). In addition to the wells for the sample extracts, there were also blanks (cell-free medium), negative control wells (cells in cell culture medium), and positive control wells (cells in cell culture medium with 10% dimethylsulfoxide). After incubation at 37 °C and 5% CO_2_ for 24 h, the extracts were used. The medium in the wells was removed with a glass pipette, and 100 µL of the extracts were added. This was followed by incubation at 37 °C and 5% CO_2_ for 24 h. For measurement with PrestoBlue (PrestoBlue™ Viability Reagent, Thermo Fisher Scientific, Waltham, MA, USA), the extracts in the wells were removed with a glass pipette. Then 90 µL RPMI and 10 µL PrestoBlue were pipetted into each well. The 96-well plates were then wrapped in aluminum foil, shaken for 5 s in a microplate spectrophotometer (Thermo Scientific Multiskan GO Microplate Spectrophotometer, Thermo Fisher Scientific, Waltham, MA, USA) at low level and then incubated for 60 min at 37 °C and 5% CO_2_. Absorbance was measured at wavelengths of 570 nm and 600 nm with the microplate spectrophotometer. A triple determination was chosen. The reduction of PrestoBlue (in %) was determined according to manufacturer’s specifications using the following formula:

(2) $$Reduction\;\left(\%\right)=\frac{(O2\;\times \;A1)\;-\;(O1\;\times \;A2)}{(R1\;\times \;N2)\;-\;(R2\;\times \;N1)\;}\times 100$$

O1 = molar extinction coefficient of oxidized PrestoBlue™ reagent at 570 nm = 80,586; O2 = molar extinction coefficient of oxidized PrestoBlue™ reagent at 600 nm = 117,216; R1 = molar extinction coefficient of reduced PrestoBlue™ reagent at 570 nm = 155,677; R2 = molar extinction coefficient of reduced PrestoBlue™ reagent at 600 nm = 14,652; A1 = absorbance of test wells at 570 nm; A2 = absorbance of test wells at 600 nm; N1 = absorbance of media only wells at 570 nm; N2 = absorbance of media only wells at 600 nm.

To investigate biocompatibility by direct contact, samples were disinfected with 70% ethanol and UV sterilized for 30 min. Then, 1 × 105 L929 cells were seeded in 100 µL of medium onto the samples in a 24-well plate. Half an hour was spent waiting. After that, each well was carefully filled with 0.5 mL of medium. After 24 h incubation at 37 °C and 5% CO_2_, the medium was removed with a glass pipette, and 100 µL of the Fluorescein diacetate (FDA) staining solution (5 mL cell culture medium without FBS, 8 µL FDA) was added to each well. The plate was wrapped in aluminum foil and incubated at room temperature for 5 min. The staining solution was then carefully removed with a glass pipette and washed once with phosphate-buffered saline (PBS). A 0.5 mL amount of Dulbecco’s phosphate-buffered saline (DPBS) was added to each well. Forceps were used to place the specimens on a microscope slide. Each specimen’s surface was then observed using a digital microscope (VHX-5000, Keyence, Osaka, Japan) with a fluorescence adapter (adapter for Keyence, Nightsea, Hatfield, PA, USA) at ×30 and ×200 magnification. In addition, scanning electron microscopy (SEM) investigation was performed after paraformaldehyde fixation, rinsing in PBS, sample dehydration in serial ethanol concentrations diluted in water, and sample storage in a desiccator for 48 h.

### 2.7. SEM Analysis

For SEM analysis, samples were sputter-coated with gold to characterize the surface topography. The surface topography was then observed by SEM (Phenom XL, Thermo Fisher Scientific, Waltham, MA, USA) at 15 kV with back scattered detector (BSD).

### 2.8. Statistical Analysis

For statistical analysis, sample size was calculated based on the pilot phase, which demonstrated the n = 7 to be sufficient in order to detect the difference of 0.07 µm in R_a_ surface roughness with an alpha 5%, a power of 80% and a standard deviation of 0.01 µm. All gathered data were statistically analyzed in a statistical software program (JMP 14, SAS Corp., Cary, NC, USA). First, the data were normalized by goodness of fit using the Shapiro–Wilk test. For normally distributed data, the statistical difference was analyzed using a two-way analysis of variance (ANOVA) with material and surface treatment as two independent factors. Tukey test was performed for multiple comparison analysis. The threshold for significance was defined as a *p*-value less than 0.05.

## 3. Results

**Figure 3:**
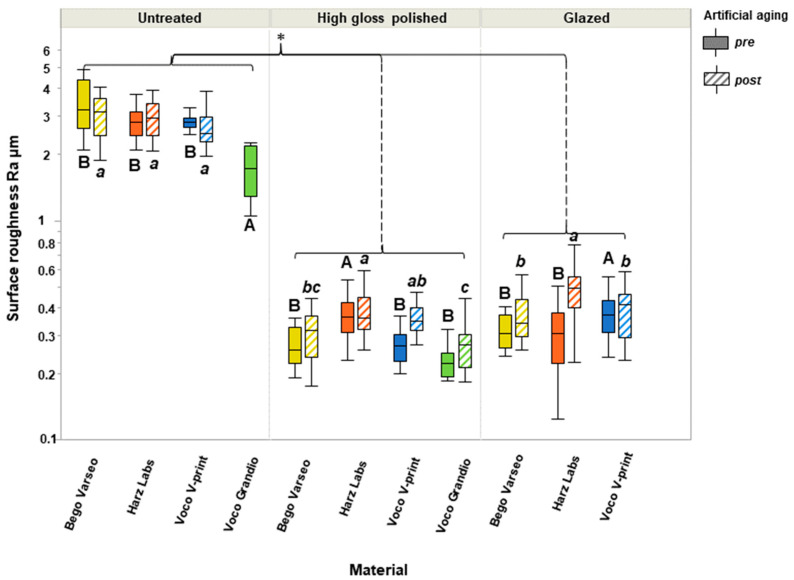
Surface roughness (Ra) values for untreated, polished, and glazed groups using four different materials. Asterisk indicates statistically significant differences between surface treatment groups using ANOVA. Letters “A, a” indicate statistical differences within each surface treatment group for various materials according to Tukey test.

Regarding surface roughness, ANOVA revealed a statistically significant difference between untreated and other groups (*p* < 0.001). For all materials, there was no significant difference before and after aging. Figure 3 illustrates the Tukey post hoc multiple comparisons between all groups.

**Figure 4:**
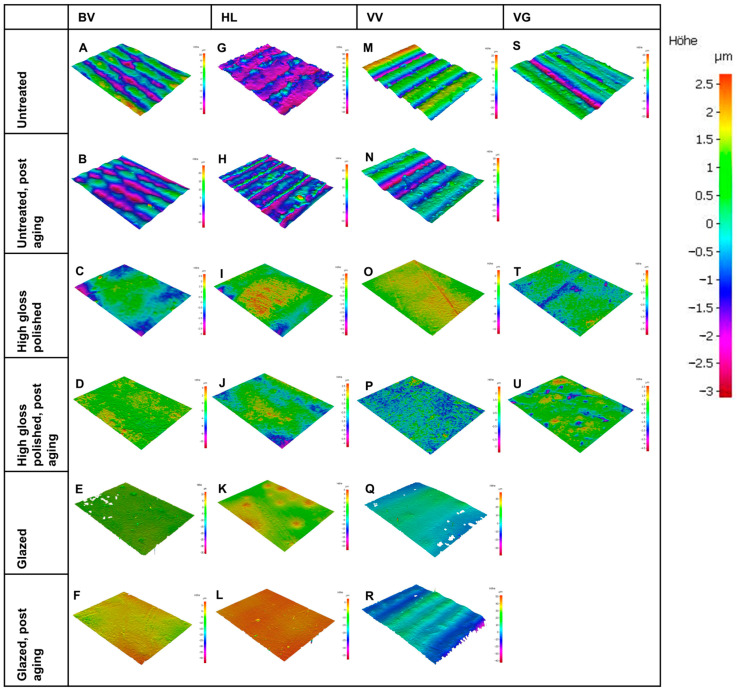
Color images as depth views from the optical roughness analysis of the samples.

In the untreated group, VG had significantly lower R_a_ values compared to 3D-printed specimens (*p* < 0.001). In the polished group, HL had significantly higher R_a_ values compared to all other groups (*p* < 0.001). This effect persisted after artificial aging. In the glazed group, VV had significantly higher roughness (*p* < 0.001), while HL had the highest roughness after aging. The color images as depth views from the optical roughness analysis of the samples are shown in Figure 4.

**Figure 5:**
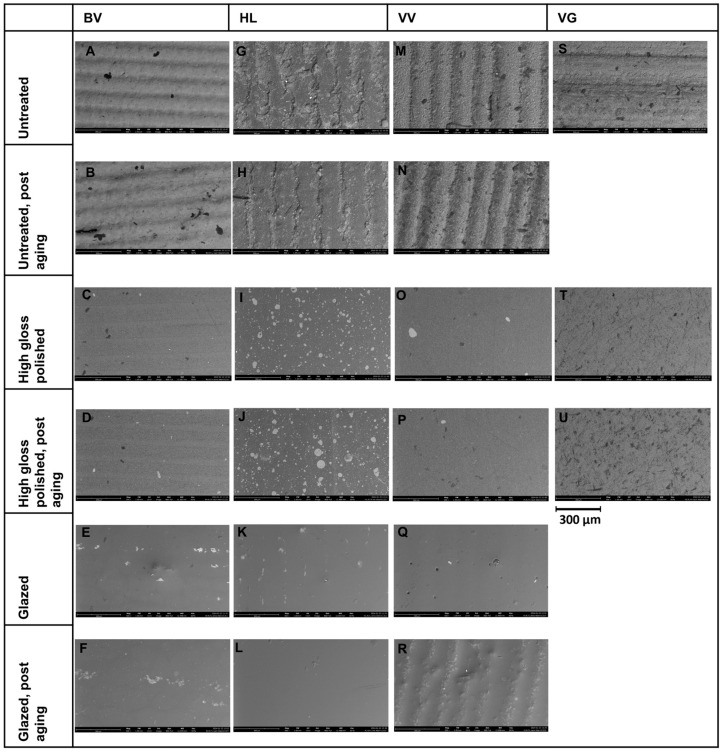
SEM images at ×500 magnification. BV group: (**A**), untreated; (**B**), untreated, post-aging; (**C**), polished; (**D**), polished, post-aging; (**E**), glazed; and (**F**), glazed, post-aging. HL group: (**G**), untreated; (**H**), untreated, post-aging; (**I**), polished; (**J**), polished, post-aging; (**K**), glazed; and (**L**), glazed, post-aging. VV group: (**M**), untreated; (**N**), untreated, post aging; (**O**), polished; (**P**), polished, post aging; (**Q**), glazed; and (**R**), glazed, post aging. VG group: (**S**), untreated; (**T**), polished; and (**U**), polished, post-aging.

SEM images (Figure 5) showed substantial differences regarding untreated, polished, and glazed surfaces. While the untreated surfaces of the 3D-printed materials showed coarse particles and repeated oblique ridges (Figure 5A,B,G,H,M,N), the untreated milled specimens were characterized by a smoother surface with many parallel-oriented fine lines (Figure 5S). The polished and glazed specimens in all groups were generally similar to each other and showed a smoother surface compared to untreated specimens. While the polished surfaces showed differently oriented patterns (Figure 5C,D,I,J,O,P,T,U), the surfaces of glazed specimens were characterized by unevenly distributed air bubbles (Figure 5E,F,K,L,Q,R).

**Figure 6:**
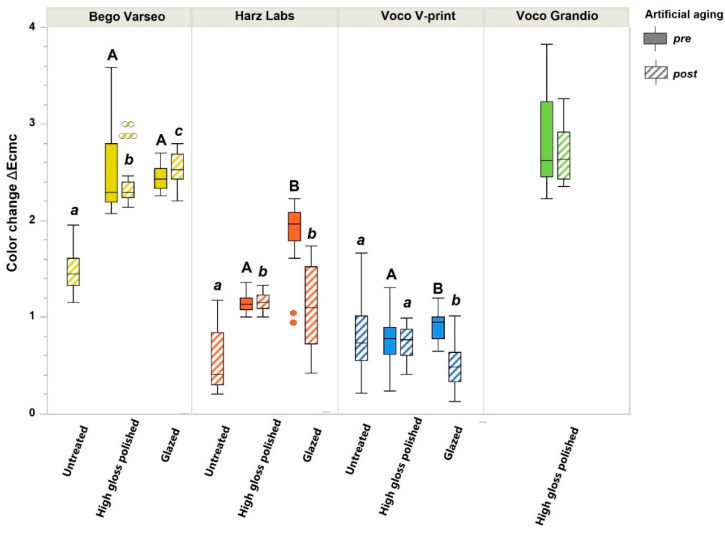
Color change (ΔEcmc) values for untreated, polished, and glazed groups using four different materials. Untreated pre-artificial aging group set as control group, providing ΔEcmc values for all measurements of the same material. Letters “A, a” indicate statistical differences within each surface treatment group for various materials according to Tukey test.

Regarding color changes, ANOVA revealed a statistically significant difference between the untreated pre-artificial aging group (reference) and the surface-treated and/or aged groups for all materials. For BV, there was no significant difference between polished and glazed groups before aging. Artificial aging of polished samples led to a significantly higher color change compared to untreated samples. This effect was even greater in the glazed group. For HL, the glazed group showed a significantly higher ΔEcmc value before aging than the polished group. After artificial aging, the polished and glazed groups demonstrated significantly higher color change values compared to the untreated group. For VV, there was a significant difference between the polished and glazed groups before aging. This effect remained the same after artificial aging, whereby there was no significant difference in color stability between the untreated and polished groups after aging. With regard to VG, artificial aging had no significant negative effect on the polished samples. Figure 6 illustrates the post hoc multiple comparisons between the groups.

**Figure 7:**
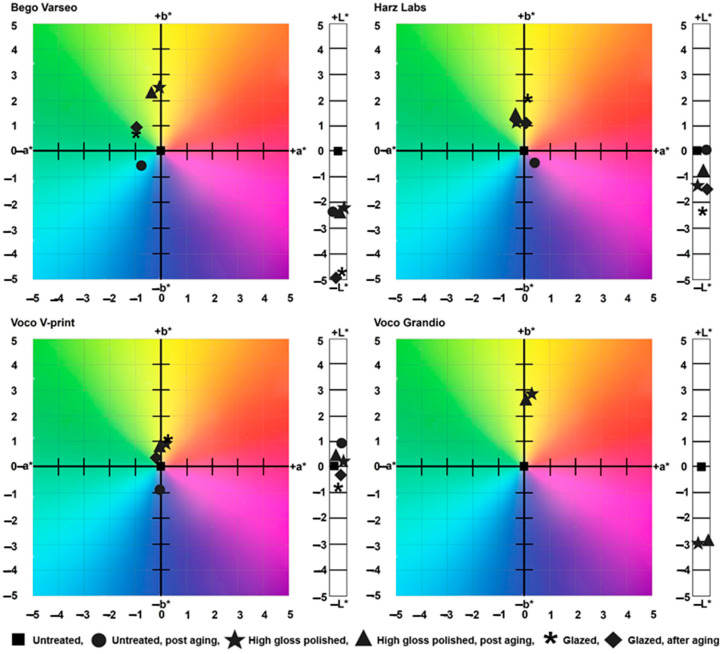
Color differences in the CIE L* a* b* color space for untreated, polished, and glazed groups using four different materials.

Figure 7 shows the L*, a*, and b* deviations of the mean value of the surface-treated and/or aged samples from the mean value of the untreated pre-aging samples in the CIE L* a* b* color space.

The mean ± standard deviation (SD) of color values in the CIE L* a* b* color space for all groups is listed in Table 4. 

**Figure 8:**
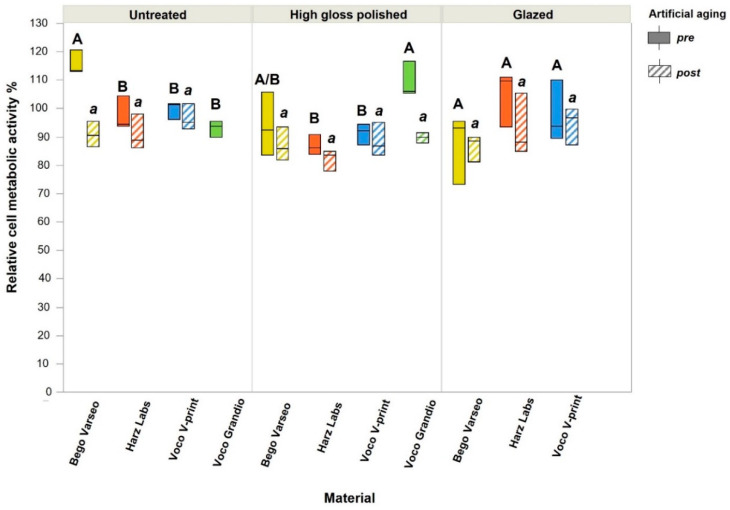
Biocompatibility values for untreated, polished, and glazed groups of four different materials relative to negative control (cells in cell culture medium), defined as 100%. Letters indicate statistical differences within each surface treatment group for various materials according to Tukey test.

Regarding biocompatibility, ANOVA revealed a statistically significant difference in artificial aging for all materials. In the untreated group, BV demonstrated significantly higher biocompatibility before artificial aging compared to all other materials. In the polished group, VG showed significantly higher biocompatibility before artificial aging compared to HL and VV. In the glazed group, there were no significant differences between the different materials. No significant differences were found between all artificially aged groups with the same surface treatment. Figure 8 illustrates the post hoc multiple comparisons between all groups.

**Figure 9:**
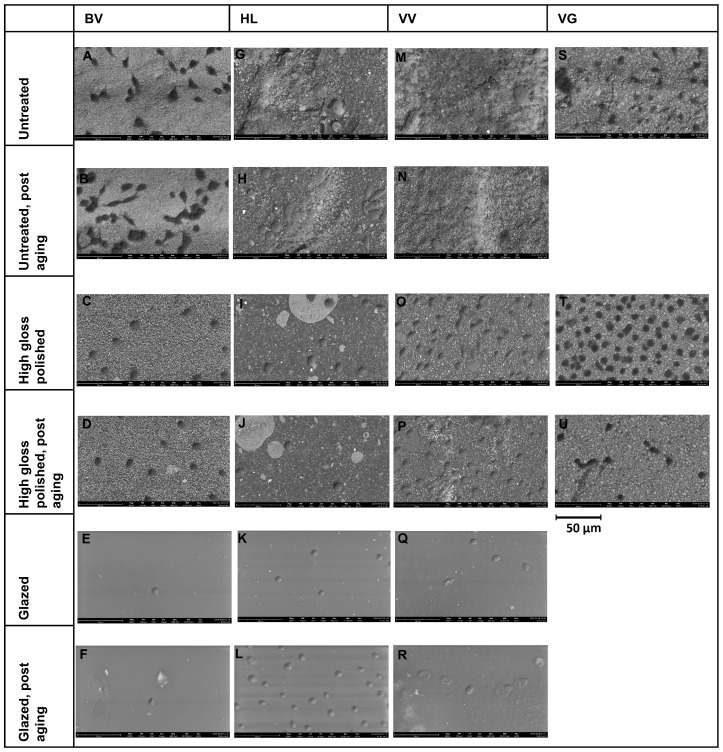
SEM images with cells at ×3000 magnification. BV group: (**A**), untreated; (**B**), untreated, post-aging; (**C**), polished; (**D**), polished, post-aging; (**E**), glazed; and (**F**), glazed, post-aging. HL group: (**G**), untreated; (**H**), untreated, post-aging; (**I**), polished; (**J**), polished, post-aging; (**K**), glazed; and (**L**), glazed, post-aging. VV group: (**M**), untreated; (**N**), untreated, post-aging; (**O**), polished; (**P**), polished, post-aging; (**Q**), glazed; and (**R**), glazed, post-aging. VG group: (**S**), untreated; (**T**), polished; and (**U**), polished, post-aging.

Compared to the negative control (cells in cell culture medium), no group showed cytotoxicity according to DIN EN ISO 10993-05 [22]. Figure 9 shows representative SEM images of the specimen’s surfaces with cells.

Cells were found on every sample of the materials examined. However, it appears that cell adhesion was best on the untreated BV surface (Figure 9A,B) and on milled, polished VG surfaces (Figure 9T).

**Figure 10:**
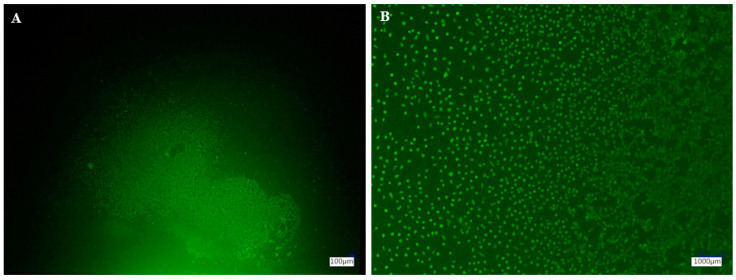
Keyence images of FDA-stained cells on polished VG specimen: (**A**), at ×30 magnification and (**B**), at ×200 magnification.

These observations were also made using the Keyence (Figure 10).

## 4. Discussion

Significant differences between various materials and treatment protocols were observed in this study. Thus, the null hypothesis was rejected. The results of the statistical analysis indicated that the R_a_ values were significantly affected by the surface treatment. In this study, specimens of the four hybrid resin-ceramic materials with untreated surfaces had the highest R_a_ value. The milled material VG had significantly lower R_a_ values than 3D-printed specimens when untreated. Other studies have reported similar results [9,23,24]. Regarding the different surface roughness values of untreated SM and AM samples, Prause et al. [25] recently investigated the microstructure of resin-ceramic materials. Using micro-computed tomography and SEM, they observed that a milled hybrid material had a more homogeneous filler distribution than a 3D-printed hybrid material, which had agglomerations, a layered macrostructure, and spherical pores due to layerwise polymerization [25]. It has been reported that the higher, more homogeneous filler content and small particle size of milled hybrid materials may be advantageous in terms of mechanical properties compared to DLP-printed hybrid materials [6]. The favorable low surface roughness of VG compared to the 3D-printed resin-ceramic materials could, therefore, be related to the particular properties of VG, namely the higher proportion of inorganic fillers (86 wt%), the small particle size and the more homogeneous distribution of the components [6]. In this study, there was no significant difference between the R_a_ values of polished and glazed groups for all materials before and after artificial aging. All polished and glazed specimens had significantly lower R_a_ values than untreated specimens, making these treatments recommended to extend restoration life and reduce the adsorption of stains and plaque accumulation [26]. These findings were confirmed by an SEM examination. Untreated specimens of DLP-printed materials had coarse particles and ridges, which refer to the layer-by-layer printing process [18,25]. SEM images of untreated milled specimens indicated a smoother surface with parallel-oriented fine lines caused by the milling cutter [18]. Polished and glazed specimens had a smoother surface compared to specimens with untreated surfaces. The glazed specimens had unevenly distributed air bubbles. In this study, there was no correlation between surface roughness and color stability of the hybrid resin-ceramic materials. Color values differed significantly between untreated and treated/aged groups. Other studies have also reported an influence of surface treatment and material type on the ΔE* values [27]. Assuming, as previously reported [27,28], that the clinically acceptable ΔE* value for color change in dental restorative materials is less than approximately 3.3, all groups in this study showed acceptable color stability. This aligns with Krajangta et al. [29], who found an acceptable color change for printed and milled hybrid resin-ceramic materials after 30 days of water immersion. Another study found that the marginal adaptation of hybrid resin-ceramic restorations, as an important parameter for the development of secondary caries and stain retention, was comparable to that of lithium disilicate, both before and after artificial aging [30]. Aging in liquids leads to water absorption of the porous resin-ceramic materials, monomer elution, and, as a result of this change in the microstructure, an altered light interaction, visible as color change [29].

The in vitro results of this study may show a trend that has been confirmed in vivo [31]. Clinical use of 3D-printed resin-ceramic restorations for 12 months yielded promising results in terms of color stability, with most cases showing no obvious color changes [31]. No biological complications occurred in any restoration (n = 171), and technical complications were rare [31].

Biocompatibility was measured by the direct method and the indirect method with sample extracts. While BV showed the significantly highest biocompatibility in the untreated group and VG showed significantly higher biocompatibility than HL and VV in the polished group, no differences were found between the different materials in the glazed group before artificial aging. Artificial aging reduced the biocompatibility of all materials, but no statistically relevant differences were found between the aged materials within all treatment groups. According to DIN EN ISO 10993-05, the test item has a cytotoxic potential if the cell viability is reduced to <70% of the blank test (cells with cell culture medium). No group showed cytotoxicity. The biocompatibility assessment using the direct method with staining and subsequent SEM analysis also showed that L929 cells adhered to the surfaces of all materials tested. Different cell morphologies are recognizable. While cell spreading indicates good adherence, cell rounding, and limited cell adhesion are signs of cell death [32]. One explanation for the observation that untreated BV surfaces showed particularly good cell adhesion in this study could be the finding from a previous study that rougher surfaces show the best cell adhesion and proliferation [33]. Changes in hydrophilicity or hydrophobicity due to different surface treatments may also affect cell adhesion [34]. As we have not investigated this in detail, further studies can focus on this aspect. We also did not test the immersion of samples in different staining solutions, so only the effects of the treatment protocol were tested.

This study clearly results in a recommendation for surface treatment, especially for high-gloss polishing of the fixed dental restoration made of hybrid resin-ceramic material, as a smoother surface can be achieved compared to glazing. If the surface roughness of the dental restoration is low, stains and plaque are less likely to adhere, which consequently reduces the risk of discoloration of the crown/bridge as well as the risk of gingivitis and secondary caries for the patient [14,26]. Although it can be argued that glazing might be a time-saving method for finishing the surface of dental restoration, our study showed that both mechanical polishing and glazing, can be considered to be a viable treatment option, as all tested materials demonstrated good biological properties in every surface condition, with no expected cytotoxic effects for the patient.

Limitations of this study included that all specimens were printed with only one printing orientation (20 degrees), as it has been described that the printing direction may affect material properties and potentially also color stability [25]. Further investigations should take this into account. In addition, tests were not performed in an environment that replicates the real oral environment; thus, a wide variety of clinical factors could influence the surface roughness, color stability, and biocompatibility of hybrid resin-ceramic restorations. Clinical studies are needed to determine if these findings align with clinical practice.

## 5. Conclusions

Based on the results of this in vitro study, also considering its limitations, the following conclusions were drawn:

1. In an untreated state, SM has an advantage over AM in terms of surface roughness. However, polishing and coating reduce the surface roughness, making it comparable to the SM group.
2. Color changes occur for all materials due to surface treatment, with glazing causing a higher color change than mechanical polishing.
3. All tested materials demonstrate acceptable biocompatibility.
4. Artificial aging of 5000 thermal cycles affects color stability and biocompatibility but not surface roughness. All materials show acceptable color changes and no cytotoxicity.

## Figures and Tables

**Table 1:** Investigated CAD/CAM hybrid materials and glazing materials of this study. Source: manufacturer’s material safety data sheets, instructions for use, and Mao et al. [17].

Manufacturing Method	Material (Manufacturer)	Code	Composition
DLP printing	VarseoSmile Crown plus A3 (BEGO, Bremen, Germany)	BV	Ceramic-filled (30–50 wt% inorganic fillers; particle size 0.7 μm) silanized dental glass, methyl benzoylformate, diphenyl (2,4,6-trimethylbenzoyl) phosphine oxide hybrid material
DLP printing	HARZ Labs Dental Sand Pro A1-A2 (HARZ Labs, Riga, Latvia)	HL	Ceramic-filled composite resin with urethane methacrylate oligomer (50–70%), reactive diluent (30–50%), 2-hydroxypropyl methacrylate (1–5%), diphenyl(2,4,6-trimethylbenzoyl)phosphine oxide (1–3%), filler (10–30%)
DLP printing	Voco V-Print c&b temp A3 (VOCO, Cuxhaven, Germany)	VV	Ceramic-filled (26 wt% inorganic fillers) hybrid material with aliphatic urethane dimethacrylate (>10–25%), aliphatic acrylate (>2.5–10%), triethylene glycol dimethacrylate (>2.5–10%), diphenyl(2,4,6- trimethylbenzoyl)phosphine oxide (max. 2.5%)
Milling	Voco Grandio blocs A3 LT (VOCO, Cuxhaven, Germany)	VG	Resin nanohybrid composite (86 wt% inorganic fillers), embedded in a polymer matrix (14% urethane dimethacrylate + dimethacrylate)
	Optiglaze Color Transparent (GC Corp., Tokyo, Japan)		Methyl methacrylate (25 ≤ 50%), silicon dioxide (5 ≤ 10%), diphenyl(2,4,6-trimethylbenzoyl)phosphine oxide (3 ≤ 5%)
	HARZ Labs Glaze (HARZ Labs, Riga, Latvia)		Urethane methacrylate oligomer (10–40%), vinyl monomer (10–15%), diphenyl(2,4,6-trimethylbenzoyl)phosphine oxide (1–5%), solvent (5–40%)
	Easy Glaze (VOCO, Cuxhaven, Germany)		Dipenta-erythritol pentaacrylat (50–100%), methyl methacrylate (25–50%), initiators (2.5–5%)

**Table 2:** Manufacturing and post-processing methods.

Material (Code)	Machine	Printing Orientation	Settings	Post-Processing
BEGO Varseo Smile Crown ^plus^ A3 (BV)	Varseo XS, BEGO, Bremen, Germany (additive)	Angulation of 20 degrees on build platform using slicing software BEGO CAMcreator Print, Version 1.32, Bremen, Germany	50 µm print layer thickness, 9 specimens per print job	Cleaning: ultrasonic bath with ethanol (96%): 3 min in reusable solution, then 2 min in fresh solution; Drying: in compressed air; removal of support structures with a cutting wheel; Sandblasting: Perlablast Micro at maximum blasting pressure of 1.5 bar; Postcuring: light curing unit (Otoflash; BEGO) for 2 × 1500 flashes, turning specimens between exposure cycles
HARZ Labs Dental Sand Pro A1-A2 (HL)	3Demax, DMG, Hamburg, Germany (additive)	Angulation of 20 degrees on build platform using nesting software Netfabb Basic 2022.0, Autodesk	50 µm print layer thickness, 40 specimens per print job	Cleaning: ultrasonic bath with isopropanol (99%) twice for 3 min each; Drying: in compressed air; Postcuring: light chamber (FormCure; Formlabs), preheated to 70 °C for 20 min; Removal of support structures
Voco V-Print c&b temp A3 (VV)	W2P-Solflex 163 Full HD, W2P, Vienna, Austria (additive)	Angulation of 20 degrees on build platform using slicing software Netfabb Basic 2023.1, Autodesk	50 µm print layer thickness, 15 specimens per print job	Cleaning: with a brush soaked in isopropanol (99%); Drying: in compressed air; removal of support structures; drying for 15 min; Postcuring: light curing unit (Otoflash, BEGO, Bremen, Germany) with 2 × 2000 flashes with a 2 min break in between
Voco Grandio blocs A3 LT (VG)	N4, vhf camfacture, Ammerbuch, Germany (milling)		Wet conditions, 2 samples milled from each block (size 14 L)	No post-processing

**Table 3:** Number of specimens for each group in this study.

		Roughness and Color Stability	Biocompatibility (Indirect)	Biocompatibility (Direct)
Additive manufacturing (BEGO VarseoSmile Crown ^plus^ A3: n = 66 Harz labs Model Sand Pro A1–2: n = 66 Voco V-Print c&b temp A3: n = 66)	Untreated, no aging	n = 7	n = 2	n = 2
	Untreated, after aging	n = 7	n = 2	n = 2
	Polished, no aging	n = 7	n = 2	n = 2
	Polished, after aging	n = 7	n = 2	n = 2
	Glazed, no aging	n = 7	n = 2	n = 2
	Glazed, after aging	n = 7	n = 2	n = 2
Subtractive manufacturing (Voco Grandio Blocs A3 LT: n = 27)	Untreated, no aging	n = 5	n = 2	n = 2
	Polished, no aging	n = 5	n = 2	n = 2
	Polished, after aging	n = 5	n = 2	n = 2

**Table 4:** Mean ± standard deviation (SD) of color values in CIE L* a* b* color space for untreated, polished, and glazed groups using four different materials.

Material	Aging?	Color	Untreated (Mean/SD)	Polished (Mean/SD)	Glazed (Mean SD)
BV	No	L*	72.20 ± 0.90	69.94 ± 1.43	67.54 ± 0.34
		a*	2.17 ± 0.60	2.02 ± 0.76	1.19 ± 0.18
		b*	11.93 ± 1.29	14.46 ± 0.96	12.71 ± 0.55
	Yes	L*	69.87 ± 0.58	69.80 ± 0.99	67.28 ± 0.43
		a*	1.39 ± 0.13	1.77 ± 0.55	1.20 ± 0.15
		b*	11.37 ± 0.61	14.27 ± 0.63	12.92 ± 0.38
HL	No	L*	74.65 ± 0.38	73.29 ± 0.31	72.28 ± 0.34
		a*	−2.69 ± 0.10	−3.02 ± 0.09	−2.61 ± 0.07
		b*	11.56 ± 0.42	12.82 ± 0.16	13.65 ± 0.38
	Yes	L*	74.71 ± 0.56	73.94 ± 0.22	73.22 ± 0.35
		a*	−2.37 ± 0.15	−3.07 ± 0.04	−2.66 ± 0.11
		b*	11.15 ± 0.44	12.97 ± 0.14	12.70 ± 0.70
VV	No	L*	66.59 ± 0.48	66.88 ± 0.22	65.79 ± 0.22
		a*	0.75 ± 0.17	0.95 ± 0.16	1.00 ± 0.15
		b*	12.36 ± 0.37	13.25 ± 0.33	13.39 ± 0.20
	Yes	L*	67.51 ± 0.45	67.05 ± 0.38	66.22 ± 0.46
		a*	0.66 ± 0.10	0.68 ± 0.27	0.53 ± 0.15
		b*	11.52 ± 0.54	13.08 ± 0.45	12.69 ± 0.34
VG	No	L*	71.77 ± 0.42	68.78 ± 0.43	
		a*	1.23 ± 0.10	1.50 ± 0.12	
		b*	7.75 ± 0.58	10.58 ± 0.55	
	Yes	L*		68.97 ± 0.26	
		a*		1.32 ± 0.10	
		b*		10.40 ± 0.31	

## Data Availability

The original contributions presented in the study are included in the article, further inquiries can be directed to the corresponding author.
